# Blockchain-Based Solution for Distribution and Delivery of COVID-19 Vaccines

**DOI:** 10.1109/ACCESS.2021.3079197

**Published:** 2021-05-11

**Authors:** Ahmad Musamih, Raja Jayaraman, Khaled Salah, Haya R. Hasan, Ibrar Yaqoob, Yousof Al-Hammadi

**Affiliations:** 1 Department of Industrial and Systems EngineeringKhalifa University of Science and Technology 105955 Abu Dhabi United Arab Emirates; 2 Department of Electrical Engineering and Computer ScienceKhalifa University of Science and Technology 105955 Abu Dhabi United Arab Emirates

**Keywords:** COVID-19 vaccines, traceability, vaccines’ distribution and delivery, Ethereum, security, smart contracts

## Abstract

Distribution and delivery of Coronavirus 2019 (COVID-19) vaccines have become challenging after their emergence. Today’s platforms and systems leveraged for managing data related to COVID-19 vaccines’ distribution and delivery fall short in providing transparency, trackability and traceability, immutability, audit, and trust features. Also, they are vulnerable to the single point of failure problem due to centralization. Such limitations hindering the safe, secure, transparent, trustworthy, and reliable distribution and delivery process of COVID-19 vaccines. In this paper, we propose an Ethereum blockchain-based solution for managing data related to COVID-19 vaccines’ distribution and delivery. We develop smart contracts to automate the traceability of COVID-19 vaccines while ensuring data provenance, transparency, security, and accountability. We integrate the Ethereum blockchain with off-chain storage to manage non-critical and large-sized data. We present algorithms and discuss their full implementation, testing, and validation details. We evaluate the proposed solution by performing cost and security analysis as well as comparing it with the existing non-blockchain and blockchain-based solutions. Performance evaluation results reveal that the proposed solution is low-cost, and our smart contracts are secure enough against possible attacks and vulnerabilities. The smart contracts code along with testing scripts is made publicly available.

## I. Introduction

Coronaviruses are human and animal pathogens. The novel Coronavirus 2019 (COVID-19) was first found in Wuhan, China. The rapid spread of this virus has resulted in a global pandemic. The World Health Organization (WHO) has designated the disease COVID-19 in February 2020 and declared it a pandemic because it was affecting an exponentially high proportion of the population [1]. COVID-19 is caused by a virus called severe acute respiratory syndrome coronavirus 2 (SARS-CoV-2).

Immunization is a key component of healthcare and is also considered a human right. Investing in immunization is extremely important and guarantees great returns. Moreover, vaccines are essential to the prevention and control of infectious disease outbreaks. Effective and safe vaccines can constitute a very powerful tool in preventing COVID-19 cases and end the pandemic. Many countries and organizations around the world are currently collaborating in the research and development of a COVID-19 vaccine, and there are already several available COVID-19 vaccines such as Pfizer-BioNTech vaccine and Moderna’s vaccine [2], [3].

A worldwide immunization campaign is very crucial to control the COVID-19 pandemic. This campaign involves many different aspects such as COVID-19 vaccine manufacturing, delivery, and administration. The only two vaccines that are currently authorized for emergency use by the Food and Drug Administration (FDA) are Moderna and Pfizer-BioNTech vaccines. The former requires frozen storage from −25°C to −15°C, refrigerated storage from 2°C to 8°C which can only last for 30 days, and light exposure can affect the quality of the vaccine. On the other hand, the latter requires frozen storage from −80°C to −60°C, refrigerated storage from 2°C to 8°C which should last only for a maximum of 5 days, and light exposure can affect the quality of the vaccine [3], [4]. The ultra-low storage temperature and the limited number of storage days indicate that the delivery process of the COVID-19 vaccines is very tedious and challenging. Such conditions require using an end-to-end cold supply chain where the vaccines are monitored from the moment they are manufactured until their administration to beneficiaries.

**FIGURE 1.**
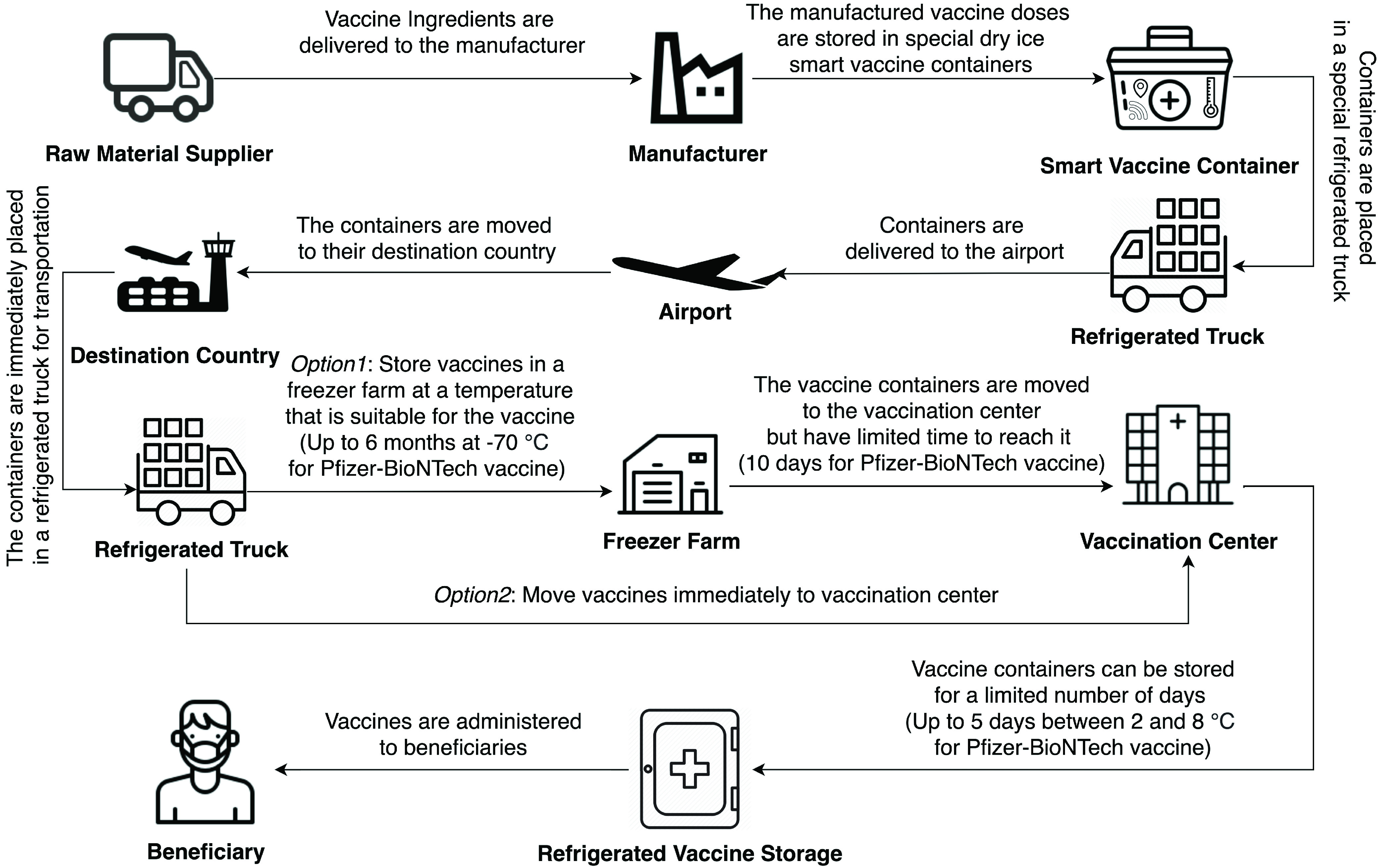
Flow diagram for the delivery of COVID-19 vaccines [5].

Figure 1 illustrates a comprehensive sequence diagram of the path that the COVID-19 vaccine (Pfizer-BioNTech) follows throughout its delivery process [5]. Note that the same process can be applied to other vaccines but the storage temperature and duration are different. First, the raw material supplier will provide the manufacturer with all the needed ingredients for the production process. Subsequently, the manufactured vaccines that are ready for delivery are stored in special dry ice smart containers that are capable of monitoring the temperature, humidity, light exposure, and the open/unopened status of the container. Then, the smart containers are placed in a refrigerated truck that immediately moves to the airport and from there they go to their destination countries. Later, the smart containers are placed again in a refrigerated truck and at this point, there are two options. In option 1, the vaccines are stored in a freezer farm which is an ultracold storage facility capable of storing millions of doses of COVID-19 vaccine and they can remain there for up to 6 months, and then they are moved to a vaccination center within 10 days from the moment they leave the freezer farm. In option 2, the vaccine containers are moved immediately to a vaccination center. Furthermore, the vaccine containers can be stored for up to 5 days in the vaccination center at a temperature between 2°C and 8°C. Finally, the vaccines are administered to beneficiaries.

COVID-19 vaccine distribution is a highly sensitive process. It requires a highly efficient solution that is capable of tracking, tracing, and monitoring the vaccines while they are being delivered. Existing efforts such as [22] and [26] only addressed the traceability aspect of the vaccines delivery. However, COVID-19 vaccines are way more sensitive than those used in the previous studies and require real-time and continuous monitoring. In this work, we propose and implement a solution that is not only capable of tracing the origin of COVID-19 vaccines, but it is also capable of monitoring the conditions of the vaccines in real-time to detect any violations while they are being delivered.

Managing COVID-19 vaccines’ supply chain requires to meet certain challenges in terms of data transparency, trackability and traceability, immutability, audit, and trust. In this context, the main contributions of this paper can be summarized as follows:

• We showcase a blockchain-based approach to enable and ensure distribution and delivery of COVID-19 vaccines in a manner that is fully trackable and traceable, transparent, auditable, reliable, trustworthy, and secure.
• We develop smart contracts to automate functionalities and generate events and notifications in case of violations to the smart vaccine container throughout the COVID-19 vaccines’ delivery and distribution process.
• We connect the Ethereum blockchain to off-chain storage to overcome the large-sized data storage limitations.
• We present algorithms and their full implementation, testing, and validation details. The smart contracts code is made publicly available.^1^
• We conduct security and cost analysis to evaluate the performance of the proposed blockchain-based solution in terms of affordability and checking robustness against possible security attacks and vulnerabilities. Also, we compare our solution with the existing non-blockchain and blockchain-based solutions.
• We propose a generic solution that can be customized as per the requirements of all types of vaccines in terms of their distribution and delivery.

^1^https://github.com/DrugTraceability/VaccineDelivery/blob/main/Code

The remainder of this paper is organized as follows. Section II presents the related work to vaccine delivery. Section III describes the proposed blockchain-based solution for the COVID-19 vaccine delivery. This is followed by the implementation of the proposed blockchain-based solution in section IV along with the details of testing and evaluation in section V. Section VI presents the discussion and analysis of the proposed solution. Section VII concludes the paper by summarizing our contributions along with future research directions.

## II. Related Work

Generally, vaccines are sensitive kinds of drugs. They are highly sensitive to exposure to heat that can directly affect public health [8]. The delivery of vaccines is usually performed using a cold supply chain wherein temperature is monitored continuously and controlled throughout storage and distribution activities [9]. The cold supply chain can be extended to other sensitive products such as fresh agricultural products, frozen food, photographic film, and pharmaceutical drugs.

In the context of cold supply chains, it is essential to differentiate between the meaning of traceability and monitoring. Traceability refers to an object whose recorded details and information are accessible throughout its life cycle. Scientifically, the object is named as Traceable Resource Unit (TRU). Traceability in supply chains can be performed in two different manners. First, it can be used to track the transaction history after the whole process is completed. On the other hand, it can be used to track the TRU in real-time [10]. Based on this definition, any tracking system will require identification techniques to distinguish TRUs from other components. Monitoring refers to the process of observing specific attributes of the TRU such as temperature, humidity, and light exposure while continuously recording them for violation detection or other analysis that might be required later on. Monitoring usually requires the installation of special sensors and equipment in the containers to transmit signals with the required information. Cold supply chains are a prime example of applications that require monitoring. For example, a sensitive product that should be delivered within a specific temperature range will require the use of monitoring so that any violation is reported instantly [11]. In the following subsection, we review the existing solutions devised to tackle the issues related to vaccines’ supply chain and their integration with blockchain.

### A. Non-Blockchain-Based Efforts for the Vaccine Cold Supply Chain

Existing efforts within the vaccine cold supply chain include reviews that discuss the current issues related to vaccines’ supply chain and how it can be resolved, technology-based solutions for the vaccine cold supply chain, and solutions that attempt to circumvent the cold supply chain completely. In 1974, the WHO established the Expanded Programme on Immunization (EPI) to establish a single and global immunization system. In 1976, the WHO presented a proposal to form a team within the EPI to address the critical issues that were preventing the WHO from establishing a global routine immunization system. The following are the three addressed issues:

• Absence of temperature monitoring systems for thermosensitive vaccines.
• Absence of equipment that are capable of storing and transporting vaccines.
• Insufficient number of trained staff that can handle the vaccines.

From the start, the WHO recognized the need for an end-to-end temperature monitoring system for vaccines in the cold supply chain. In the early 1980s, companies in the United States and Switzerland developed a cold chain monitor (CCM) which is based on blue wax absorption on a visual track [18]. Moreover, PATH (an international non-profit organization) and the WHO collaborated in the late 1970s to find a solution for tracking the heat exposure of the vaccines. PATH and the Temptime Corporation developed and commercialized a vaccine vial monitor (VVM) based on polymerization technology [19]. The WHO has made it mandatory for all the vaccines that are purchased through the United Nations Children’s Fund (UNICEF) to use VVMs. Although the VVM has solved one of the major issues in the vaccine cold supply chain, the time and location of the violation cannot be found. In addition to that, no notifications are sent to the concerned entities at the time of the violation occurred. After twenty years of the development of the CCM, the same company developed a new solution called “30DTR” which is an electronic 30-day temperature recorder [20].

Sun *et al.* [12] proposed a solution that eliminates the need for a cold supply chain to maintain the temperature of the vaccines within the acceptable range. In addition to that, Leung *et al.* [13] proposed the use of dried viral vaccines in pullulan and trehalose mixture. However, these approaches were not adopted because the pharmaceutical and logistics industries have already invested heavily in the cold supply chain [14]. Ouzayd *et al.* [15] proposed a model for instant cold supply chain monitoring using a colored Petri net (CPN) [16] which focuses on the central storage of vaccines and takes some of the WHO recommendations into considerations. Furthermore, FedEx launched its sensor technology for tracking, SenseAware ID, in November 2020, which aims to target healthcare, aerospace, and retail industries to improve their shipments data [17]. In summary, all the aforementioned attempts helped in mitigating some of the issues related to the supply chain of vaccines. However, some issues still persisted such as accountability, real-time tracking, and violation detection.

### B. Blockchain-Based Efforts in the Field of Supply Chain

Blockchain applications have been expanding rapidly since the implementation of the first blockchain (Bitcoin) which was only limited to virtual currency transactions [21]. The supply chain field is one of the fields that has been researched extensively to address its existing issues by using blockchain technology.

Siddhartha. [22] founded StaTwig platform which uses blockchain technology to connect manufacturers and users of the vaccines to increase the efficiency of the distribution process by improving traceability. One of the issues that is considered in this platform is the degradation of vaccines during their delivery because of the temperature fluctuation. Smart contracts are utilized to help mitigate these issues. Yong *et al.* [26] design a blockchain-based system for vaccine supervision in the vaccine supply chain. The smart contracts of the Ethereum blockchain were utilized to trace vaccine operations records and detect expired vaccines.

Blockchain technology has been utilized to address different issues within the supply chain industry. Tian [23] developed a traceability system for the agriculture food supply chain by combining radio-frequency identification (RFID) and blockchain technology. Andoni *et al.* [24] proposed a blockchain-based solution for controlling and tracking the energy supply. Behnke *et al.* [25] applied blockchain technology to track and trace the food supply chain. Based on the aforementioned studies, we conclude that blockchain has shown immense potential in terms of tracking and tracing supply chains. Moreover, it can play a vital role in ensuring secure, trustable, traceable, transparent, and reliable delivery of COVID-19 vaccines.

## III. A Blockchain-Based Approach for COVID-19 Vaccine Delivery

**FIGURE 2.**
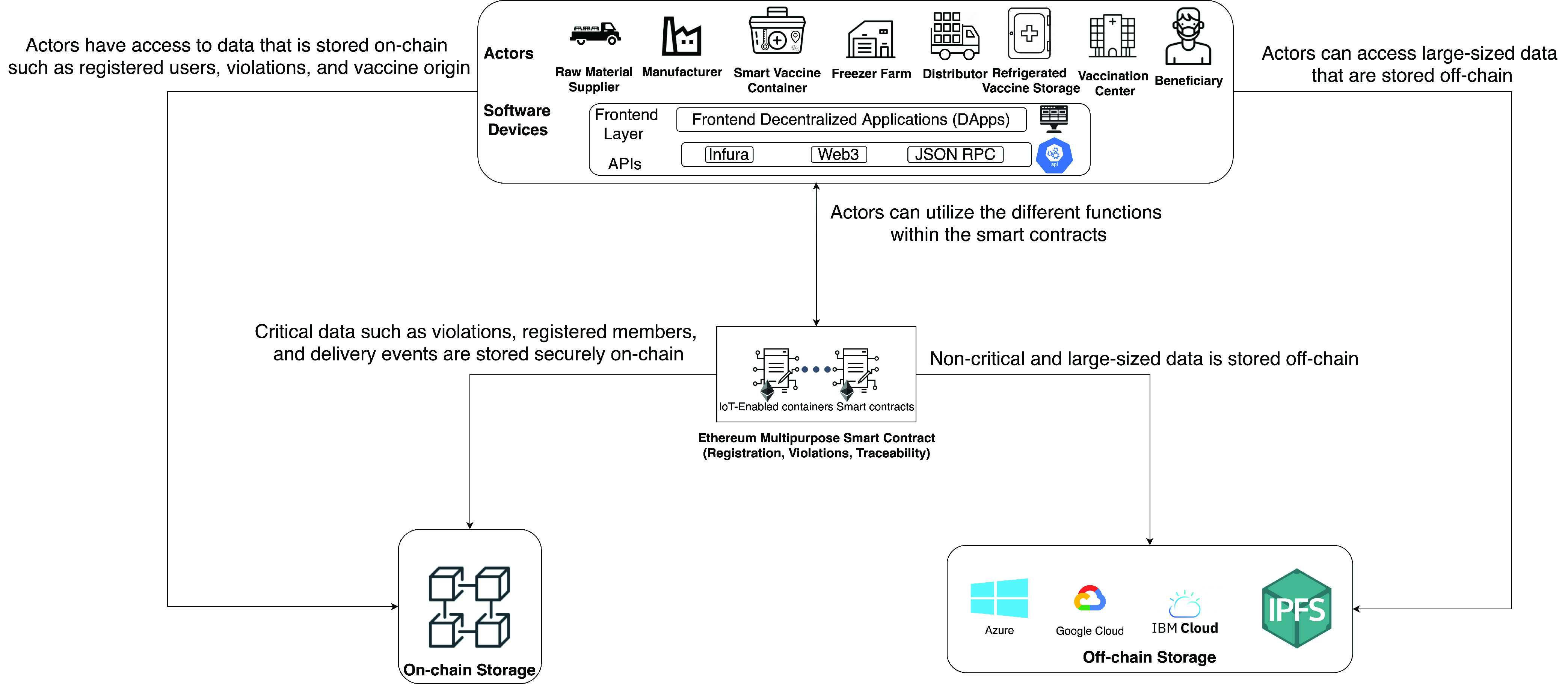
A high-level architecture for the proposed blockchain-based system for COVID-19 vaccines allocation.

In this section, we present details of our proposed blockchain-based approach for the COVID-19 vaccine delivery. Figure 2 illustrates the high-level system architecture of the proposed solution. The actors are envisaged to use Frontend Decentralized Applications (DApps) to access the registration, traceability, and violations of smart contracts. Besides, they are granted access to off-chain and on-chain storage. The software devices that are used to access the DApps will need to leverage the use of Application Program Interface (API) to link them to the smart contracts. Infura, JSON RPC, and Web3 are some examples of the available APIs. Each actor will have limited access to the smart contract functions where only pre-authorized functions are executable by the designated actor. Moreover, off-chain storage involves the handling of data that is large and would be costly to store on-chain, and for data that is non-critical throughout the delivery process. The following are the details of the system components.

• **Actors:** They include raw material suppliers, manufacturers, distributors, Internet of things (IoT)-Enabled devices (Smart vaccine container), freezer farms, refrigerated vaccine storage, vaccination centers, and beneficiaries. The actors will have pre-authorized functions in the smart contracts based on their role in the COVID-19 vaccine cold supply chain. In addition to that, the actors will be capable of viewing what is stored on-chain such as logs, transactions, and violations. Furthermore, they will have access to data that is stored off-chain.
• **Off-chain Storage:** It provides means for large data storage at a relatively low-cost compared to on-chain storage. Moreover, off-chain storage is also utilized when the data is non-critical and doesn’t need to be continuously monitored [6]. Although off-chain storage ensures low cost and the integrity of data is maintained through the hash that is stored on-chain, it still has some drawbacks. For example, if the data stored off-chain is altered, the users will detect this change because its hash will change as well; however, the original data will not be retrievable. Moreover, data loss is possible and what will only remain on-chain is the hash. Finally, additional communication mechanisms will be needed to share data off-chain [7].
• **On-chain Storage:** It is used to register actors, store logs, events generated by smart contracts, and record any violation that occurs during the delivery process of the COVID-19 vaccine smart containers which ensures reliability and provides real-time monitoring.
• **Ethereum Smart Contract:** The Ethereum smart contract has three main purposes, registration, traceability, and violations monitoring. The registration works by assigning the Ethereum address of every distributor, smart container, and vaccination center to specific roles that allow them to execute specific functions within the smart contract which is done by utilizing the Modifier feature in the Solidity language. Moreover, violations are continuously monitored during the delivery process of COVID-19 vaccine smart containers from the moment they leave their manufacturer facility until they reach a vaccination center. The violations include exceeding the temperature limits, exposure to light, the opening and closing of the container, and the route of delivery. Finally, traceability is important for tracing the origin of the vaccine to verify its authenticity. It works by triggering events and logging them onto the blockchain which are later on used by the end-user to trace back the origin of the vaccine to verify its authenticity.

The system components ensure that COVID-19 vaccines will be administered to beneficiaries in a good condition by monitoring them in real-time while they are being delivered to vaccination centers. Moreover, it allows DApp users to verify the origin of the vaccine.

The following subsections are dedicated for the detailed sequence diagram where the interactions among the actors, smart contracts, and cloud storage are presented. Also, we present the COVID-19 vaccine smart container architecture.

### A. Sequence Diagram of the Proposed Blockchain-Based Solution

**FIGURE 3.**
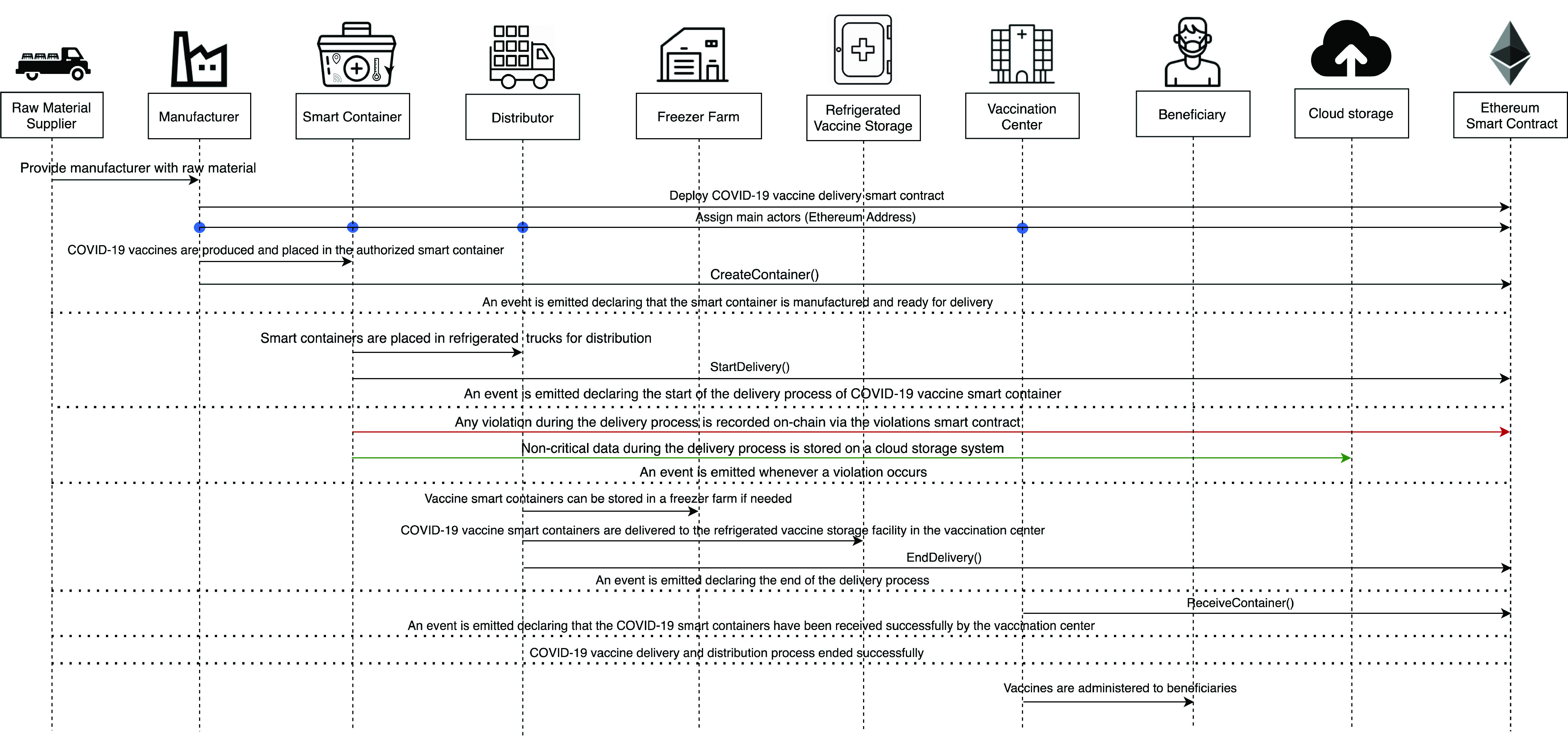
Sequence Diagram illustrating interactions among the participants of the smart contract.

Figure 3 illustrates the interactions among the different actors within the COVID-19 vaccine cold supply chain. First, the manufacturer will have to deploy the Ethereum smart contract and all the actors have to be registered via the registration function in the Ethereum Smart Contract where they will get access to specific functions by using the Modifier feature in the Solidity language. Then, the process of producing COVID-19 vaccines will begin with the delivery of raw material from the supplier to the manufacturer. After that, the manufacturer will produce the vaccines, place them in IoT-Enabled smart containers. Next, the distributor will use refrigerated trucks to transport the smart containers to either a Freezer Farm or directly to a vaccination center where the IoT-Enabled smart containers are stored in refrigerated vaccine storage. Finally, the vaccines are administered to beneficiaries. Throughout this process, all actors will be notified about any violation that occurs which are stored on-chain to sustain a high level of trust and authenticity. In addition to that, any non-critical data that is stored on the cloud storage will be accessible by the actors via the DApp. Moreover, actors will have the privilege of ensuring the origin and authenticity of vaccines by using an event-based method where an event is emitted in every stage of the delivery process and recorded on an immutable ledger.

### B. COVID-19 Vaccine Smart Container Architecture

**FIGURE 4.**
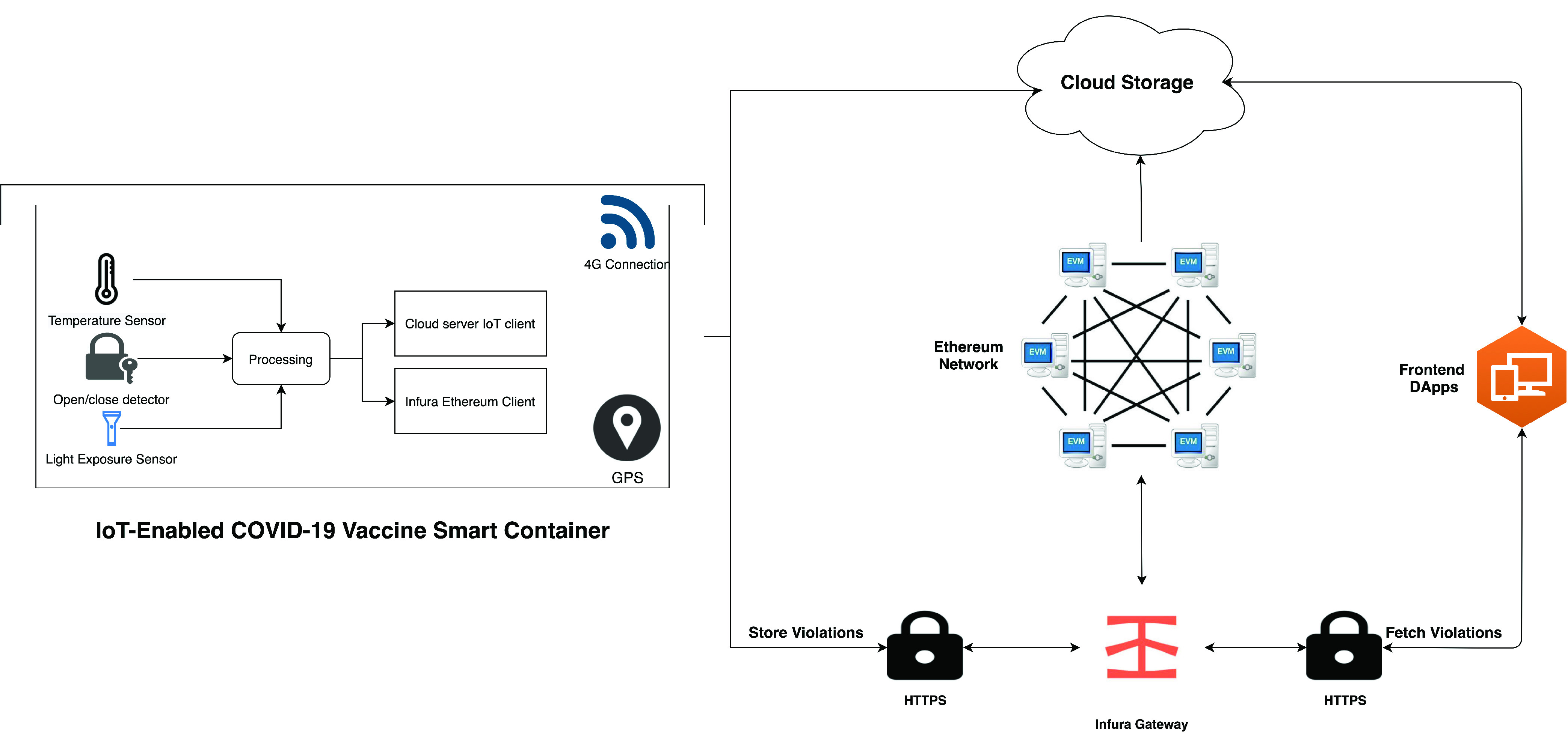
Smart container architecture for the COVID-19 vaccine.

Figure 4 illustrates the main components of the COVID-19 vaccine smart container architecture. It consists of four main components: the smart container, the cloud storage, DApps, and the Ethereum network. The diverse nature of these components requires the use of different communication media and protocols to achieve interoperability among them. The IoT-Enabled COVID-19 vaccine smart container communicates with the Ethereum blockchain to record violations and the cloud storage to record non-critical data and to provide live monitoring metrics that require large storage which would be very costly to store on the blockchain. Because the smart container is continuously on the move, a 4G connection is required to maintain its connection to the Ethereum blockchain and the cloud storage. The Infura Ethereum client is used to perform API calls between the smart container and the DApp with Infura, and thus, with the Ethereum blockchain. The DApp users will be able to notice any violations through the Ethereum smart contract.

## IV. Implementation of Proposed Solution

In this section, we present our developed algorithms and implementation details of the Ethereum smart contracts. The smart contracts are written in Solidity language. Moreover, the smart contracts are compiled and tested using REMIX IDE which is an online web-based development environment for smart contracts where codes are written and executed. In addition to that, it allows the users to debug and test their codes. Our smart contracts code is made publicly available at GitHub.^2^^2^https://github.com/DrugTraceability/VaccineDelivery/blob/main/Code

The smart contract contains multiple types of variables. Public Ethereum address which includes the manufacturer, smart container, and the vaccination center. Moreover, it has the mapping for the authorized distributors who are allowed to transport the smart containers from a location to another. In addition to that, there are two enumerate variables such as “containerStatus” and “violationType”. The former includes the different states the smart container will go through such as “NotReady”, “ReadyforDelivery”, “StartDelivery”, “onTrack”, “EndDelivery”, “ContainerReceived”, and “Violated” which is extremely important to prevent reentrancy. The latter includes violations such as “Temp” which refers to violating the temperature’s acceptable range, “Open” which indicates if the container was opened or not, “Light” which detects any violations to the acceptable light exposure range, and “Route” which refers to the violation of the track the smart container delivery should follow. The manufacturer of the COVID-19 vaccine will deploy the smart contract that represents the vaccine smart container. The smart contract will initially define the owner of the smart contract, which is the manufacturer, in this case, the start time of deploying the smart contract, the contents of the smart container, the Ethereum address of the smart container, and the vaccination center that will receive it, and the initial state of the smart container. Once the smart contract gets deployed, the registration, tracking, and violations monitoring processes begin. First, the manufacturer will need to register the trusted distributors to allow them to execute functions that require authorization later on. The tracking process consists of five main steps. First, the creation of the COVID-19 vaccine smart container where the manufacturer will announce that the smart container is manufactured and ready for delivery. After that, the distributor will have to pick up the smart container from the manufacturer and announce the start of the delivery process. Next, the arrival of the smart container at the vaccination center marks the end of the delivery process. Finally, the vaccination center needs to announce the recipients of the smart container. This tracking process is important for verifying the origin and authenticity of the vaccines. However, violations throughout the delivery process can affect the quality of the vaccines. Therefore, violation monitoring is essential for the success of COVID-19 vaccine delivery and administration. For this particular purpose, the smart container is considered as an actor and it will be sending signals to the smart contract to store and log any abnormalities or violations that occur throughout the delivery process. The violations include exceeding the acceptable temperature range, opening and closing the smart container, light exposure, and changing the route of the delivery. If a violation occurs, the state of the delivery process will change to “Violated” which means the vaccines within the smart container are no longer appropriate for administration.

**FIGURE 5.**
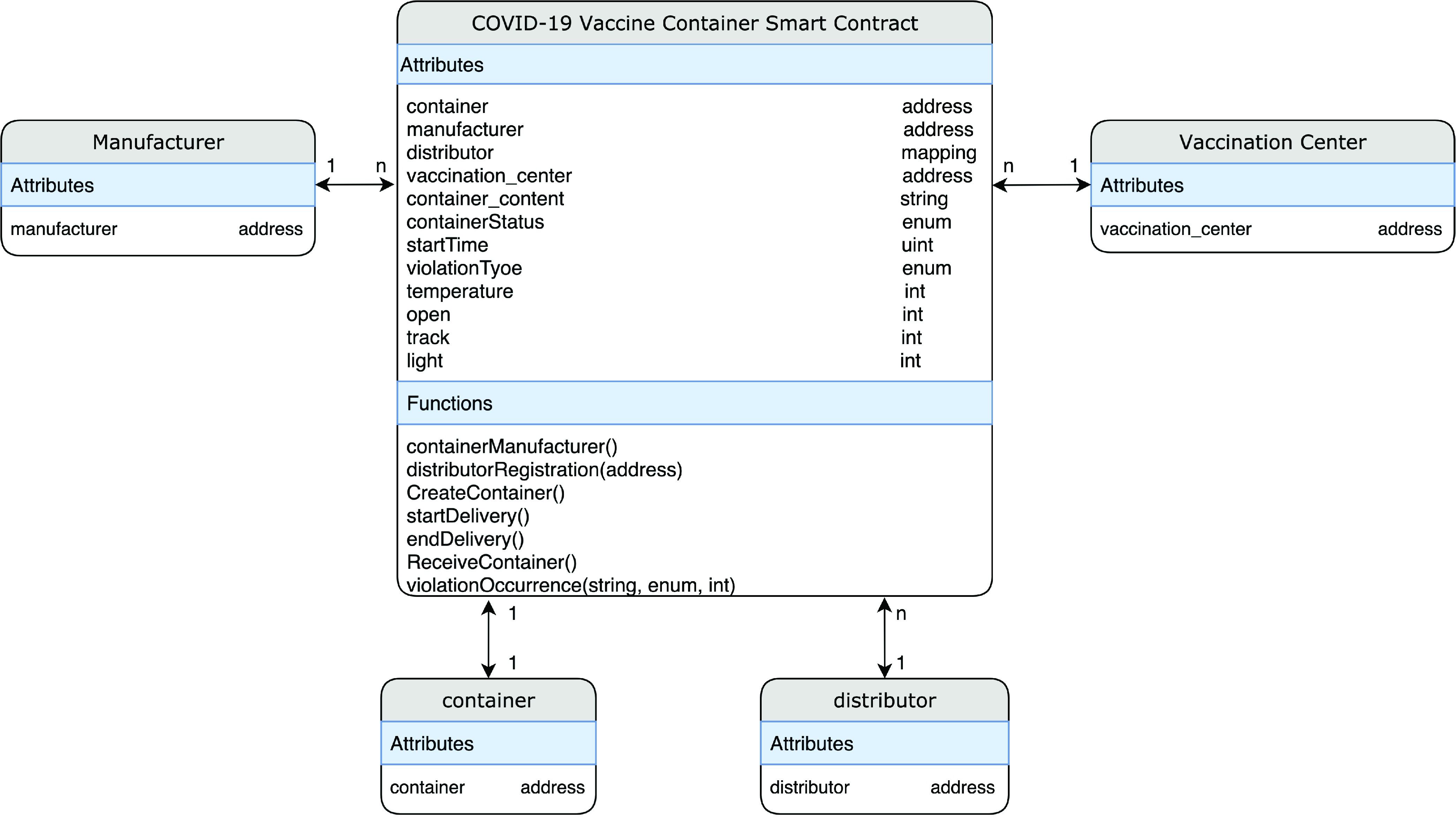
Entity-relationship diagram.

Figure 5 illustrates the entity-relationship diagram that highlights the smart contract attributes, functions, and the relationship among the involved actors in the COVID-19 vaccine delivery process. The main actors, in this case, are the Manufacturer, IoT-Enabled Smart Container, Distributor, and Vaccination Center. The manufacturer, distributor, and the vaccination center can be associated with multiple smart contracts, whereas the IoT-Enabled Smart Container can be associated with only one smart contract at a time.

**FIGURE 6.**
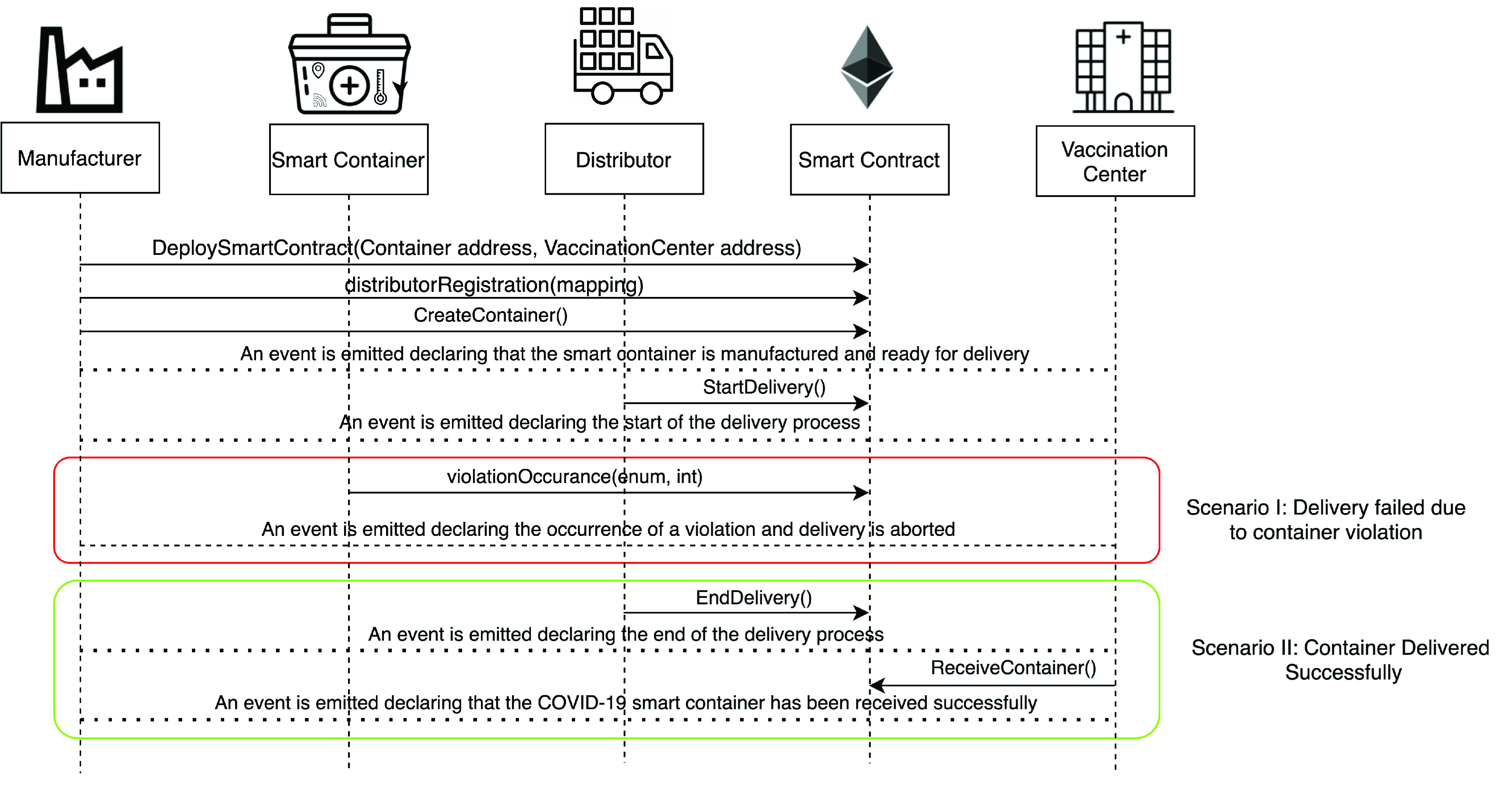
Function calls and events for two different scenarios for COVID-19 vaccine smart container delivery.

Figure 6 shows an illustrative scenario of COVID-19 vaccine smart container delivery. First, the manufacturer will deploy the smart contract where the container and vaccination center Ethereum addresses are registered to permit them to run specific functions within the smart contract. In addition to that, the manufacturer will add the eligible distributors in a mapping where the Ethereum addresses of the distributors are registered. Then, the manufacturer will announce the creation of the IoT-Enabled smart container which notifies the distributor that it’s ready for delivery. Next, the distributor will announce the start of the delivery process. After this step, there are two potential scenarios, the first is the failure of the delivery process where a violation is detected by the IoT-Enabled smart container and the delivery process is aborted, and the second scenario is when the delivery is successful and the distributor will announce the end of the delivery process, and the vaccination center will announce the recipients of the container. Seven algorithms are used to further clarify the functions of the smart contract. The following are the used functions in the smart contract along with their algorithms.

• **containerManufacturer Function**: Algorithm 1 represents the *containerManufacturer* function. This function is restricted to only read the Ethereum address of the manufacturer. It takes no input and has no modifiers, therefore, any node in the network can use it to view the manufacturer of the COVID-19 vaccine smart container.
• **distributorRegistration Function**: Algorithm 2 describes the registration process of the distributor in the smart contract to assign them their roles. The manufacturer is the only modifier of this function and hence responsible for registering authentic distributors to the network. The distributors are registered in a mapping which means a number of distributors are eligible and qualified to deliver the smart containers.
• **CreateContainer Function**: Algorithm 3 describes the function that announces the creation of the smart container. The function can be only executed by the manufacturer. Moreover, the function only works if the state of the smart container is “NotReady” meaning that the smart container has just been prepared and loaded with the vaccines for delivery.
• **StartDelivery Function**: Algorithm 4 represents the *StartDelivery* function which is only executable by a registered distributor. The function requires the state of the smart container to be “ReadyforDelivery” and once executed it emits an event declaring that the delivery process has started.
• **EndDelivery Function**: Algorithm 5 represents the *EndDelivery* function which is only executable by a registered distributor. The function requires the state of the smart container to be “onTrack” and once executed it emits an event declaring that the delivery process has ended.
• **ReceiveContainer Function**: Algorithm 6 represents the *ReceiveContainer* function which is only executable by the registered vaccination center through the constructor of the smart contract. The function requires the state of the smart container to be “EndDelivery” and once executed it emits an event declaring that the container has been received.
• **violationOccurrence Function**: Algorithm 7 represents the *violationOccurrence* function which can be only executed by the smart container when the state of the smart container is “onTrack”. There are four main violations that are monitored; namely, temperature, light, open/close, and route. The container can execute the function with two inputs such as violation type and its value. In addition to that, the function emits an event to all actors in the network declaring the type of the violation and it updates the state of the container to “Violated”.

**Algorithm 2:**
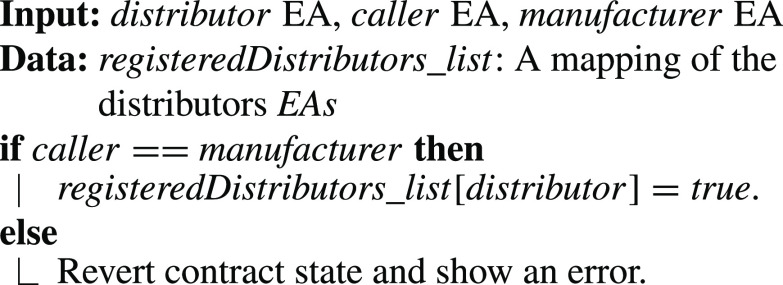
DistributorRegistration Fun

**Algorithm 3:**
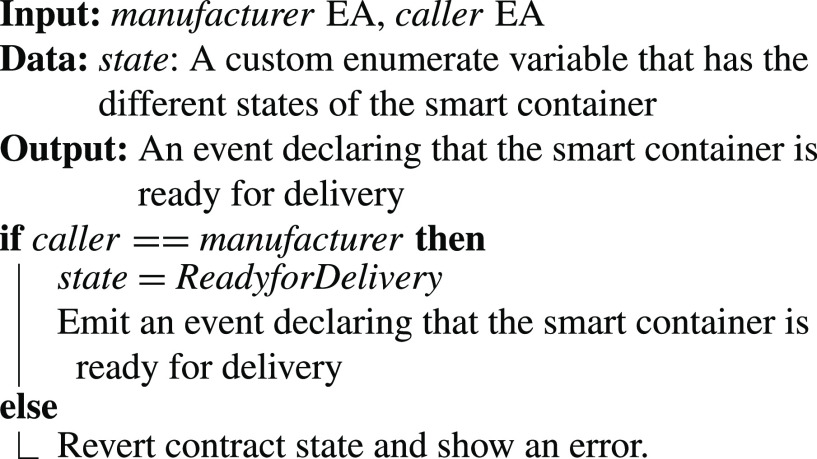
CreateContainer Function

**Algorithm 4:**
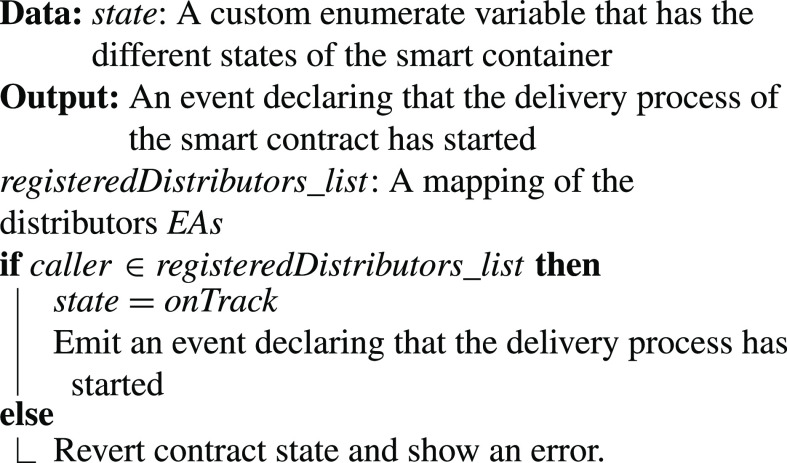
StartDelivery Function

**Algorithm 5:**
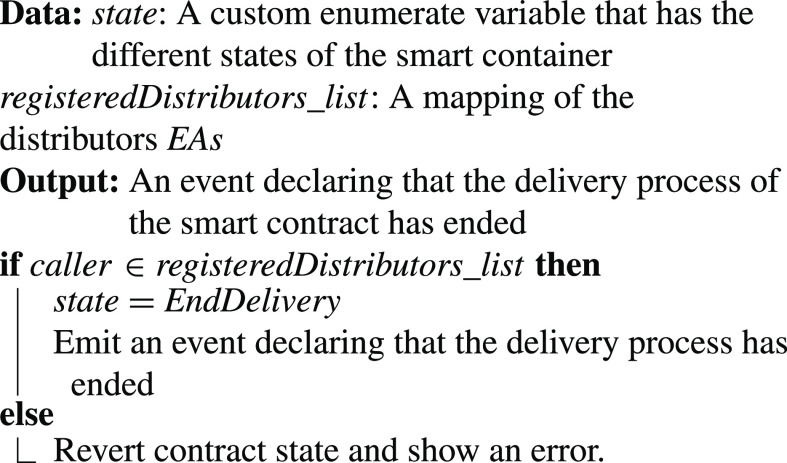
EndDelivery Function

**Algorithm 6:**
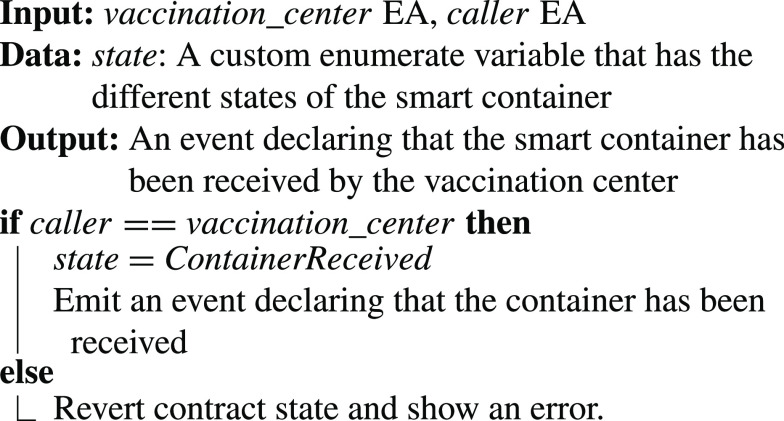
ReceiveContainer Function

**Algorithm 7:**
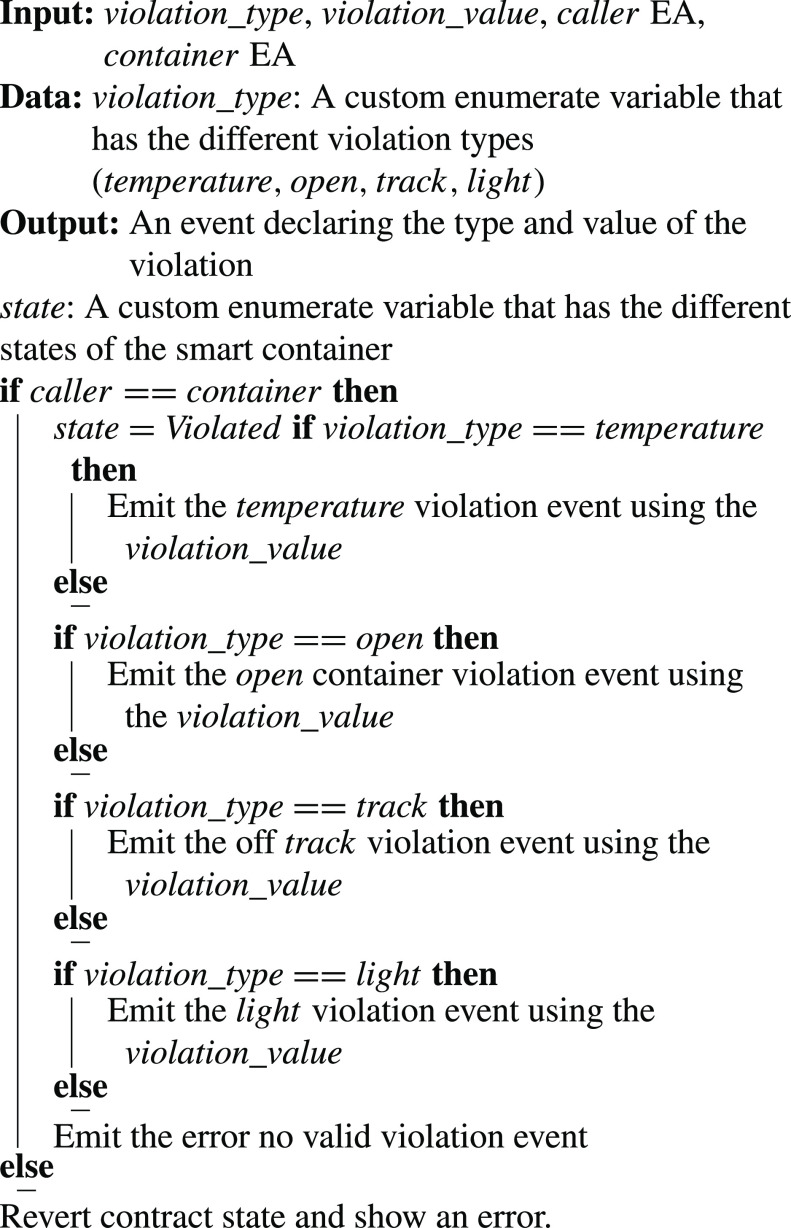
ViolationOccurrence Function

Algorithm 1: **ContainerManufacturer Function**
Output: $EA$ of the manufacturer
Return manufacturer $EA$

## V. Testing and Validation

In this section, we test and validate the developed smart contract. The assessment is performed using Remix IDE. Table 1 shows the Ethereum addresses of the different actors involved in the smart contract as they are used throughout the testing and validation process.

**TABLE 1:** The Ethereum Address of the Delivery Process Actors

Actors	Ethereum Address
Manufacturer	$0\times 5$B38Da6a701c568545dCfcB03FcB875f56beddC4
Distributor	0xAb8483F64d9C6d1EcF9b849Ae677dD3315835cb2
Container	$0\times 4$B20993Bc481177ec7E8f571ceCaE8A9e22C02db
Vaccination Center	$0\times 78731$D3Ca6b7E34aC0F824c42a7cC18A495cabaB

The transactions and logs of the smart contract’s functions are presented below.

**FIGURE 7.**
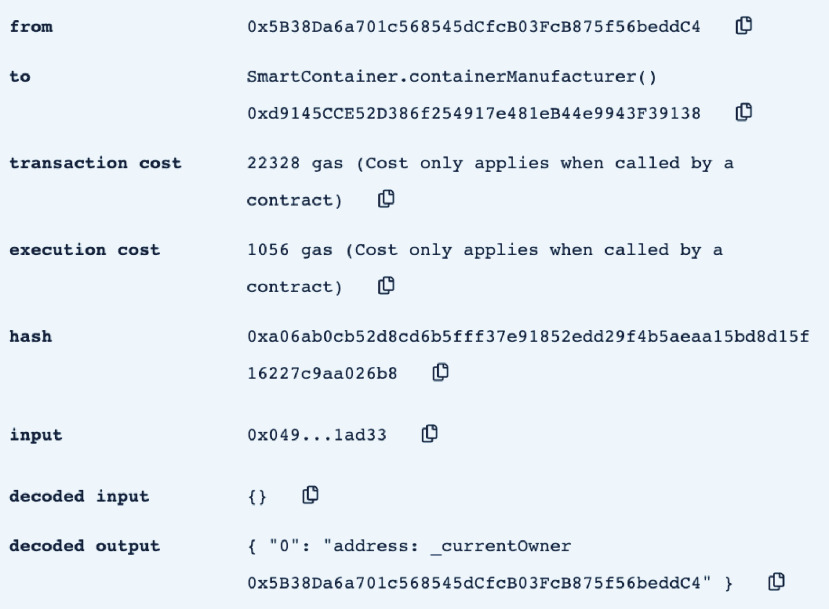
Successful execution of the containerManufacturer Function.

**FIGURE 8.**
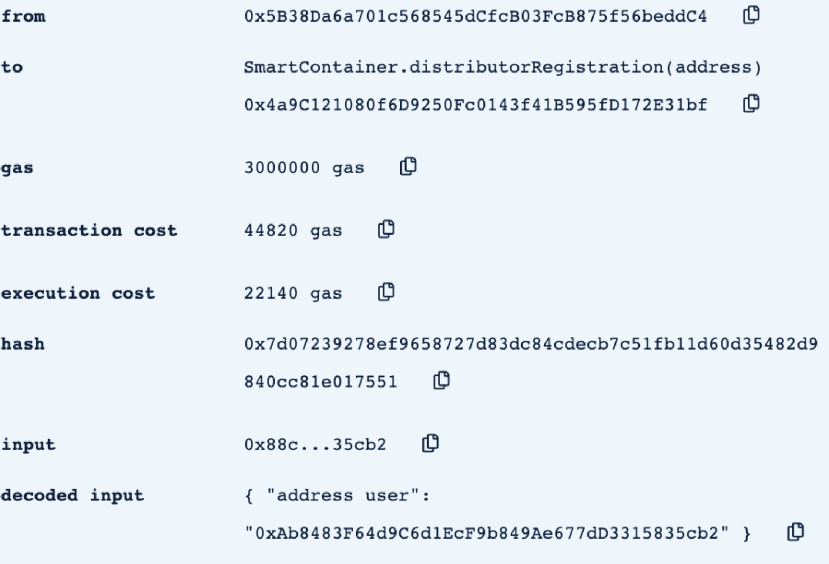
Successful execution of the distributorRegistration function Function.

**FIGURE 9.**
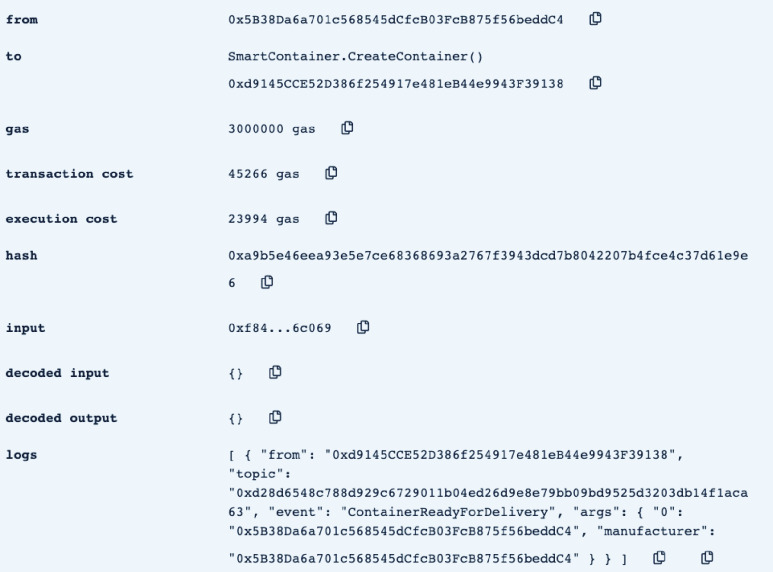
Successful execution of CreateContainer Function.

**FIGURE 10.**
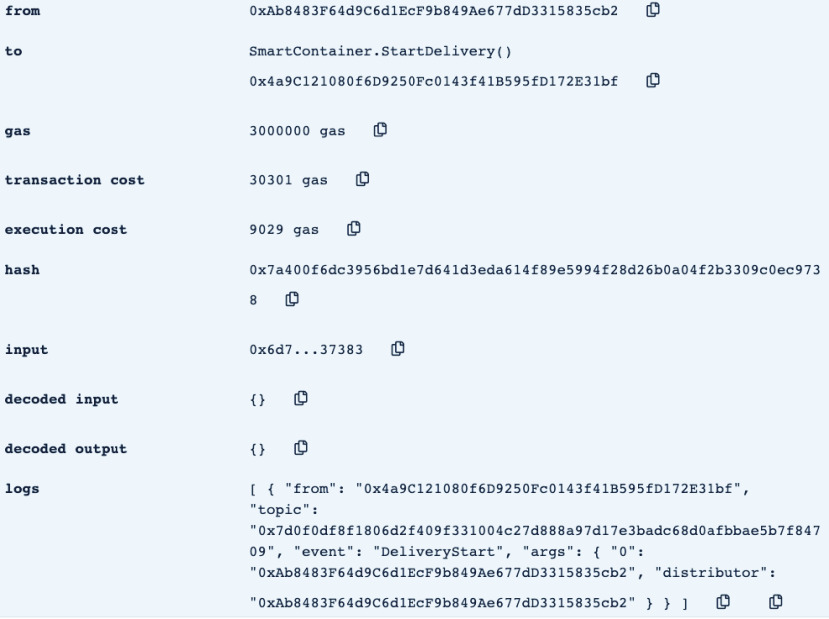
Successful execution of StartDelivery Function.

**FIGURE 11.**
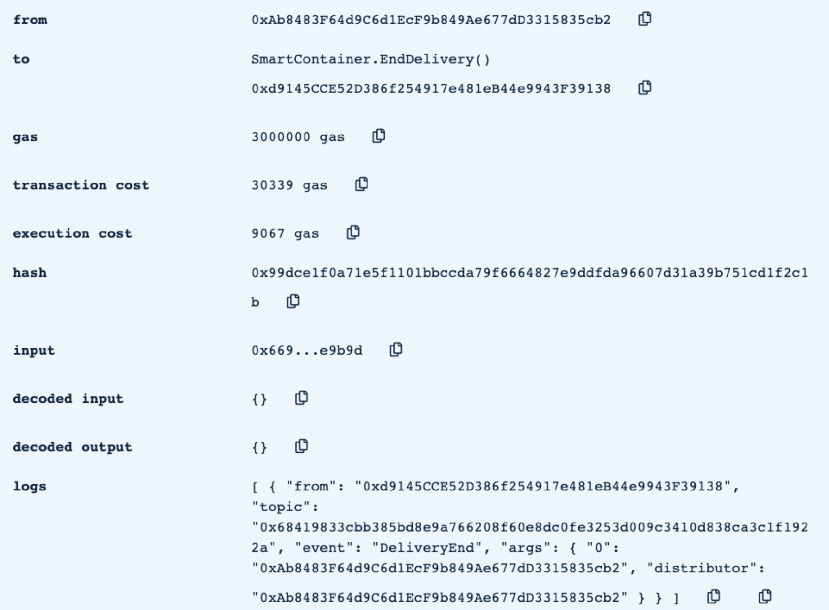
Successful execution of EndDelivery Function.

**FIGURE 12.**
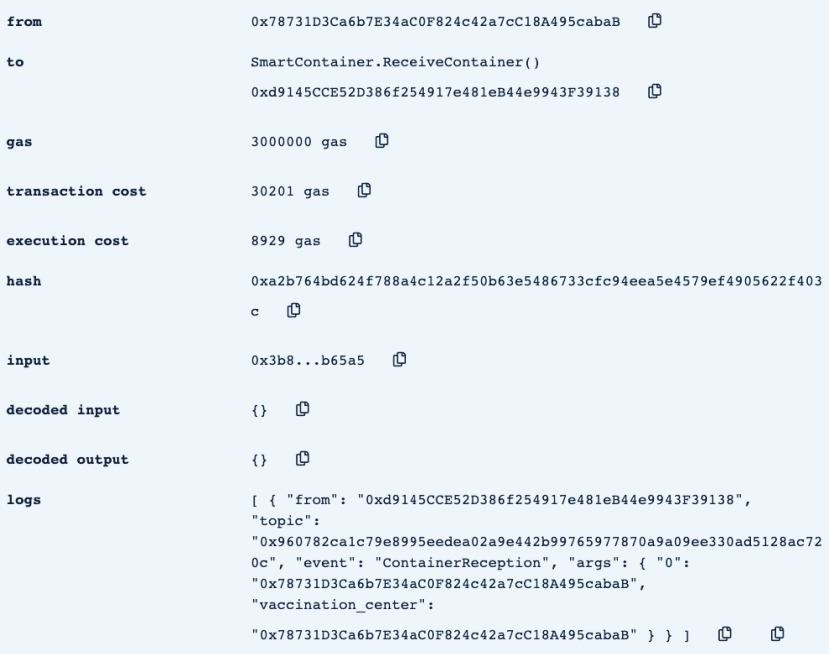
Successful execution of ReceiveContainer Function.

**FIGURE 13.**
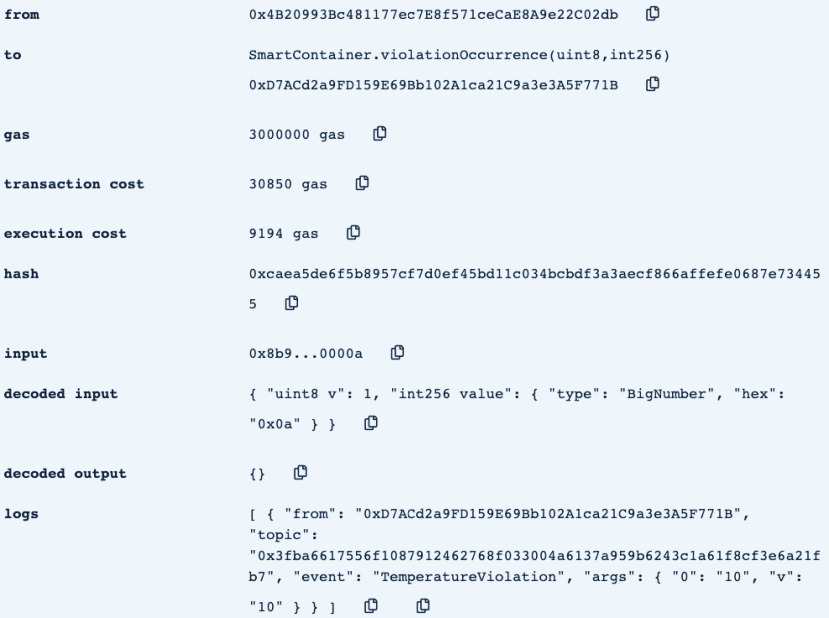
Successful execution of violationOccurrence Function.

• **containerManufacturer**: This function is tested by using a random Ethereum address in the testing network to check whether the correct manufacturer (deployer of the smart contract) would be displayed or not. Successful execution of the function and its logs and events are displayed in Figure 7. The “from” field shows the Ethereum address of the function caller who is in this case the Manufacturer and it can be verified from Table 1. Moreover, the “to” field shows the smart contract name and the function that is being called which is the *containerManufacturer* along with its address. The following fields display the gas, transaction, and execution costs respectively. In addition to that, the hash and the input of the executed function are displayed. Furthermore, the “decoded output” field shows the Ethereum address of the manufacturer which is returned to the function caller. Also, there is no decoded input in this function because its caller is not required to input anything.
• **distributorRegistration**: This function is only executable by the manufacturer where the Ethereum addresses of the trusted distributors are registered. Figure 8 shows a successful execution of the function. The “from” field shows the Ethereum address of the manufacturer who is the function caller and the “to” field shows the name and address of the called function. Moreover, gas, transaction, and execution costs are displayed respectively. In addition to that, the hash and input of the executed function are displayed in the following two fields. Furthermore, the “decoded input” field shows the Ethereum address of the distributor that is being registered and it can be verified from Table 1. Finally, there is no “decoded field” in this function because it doesn’t return anything to its executor.
• **CreateContainer**: This function is responsible for emitting an event that announces the creation of the smart container which can be only executed by the manufacturer. Figure 9 shows a successful execution of the function along with its logs and events. The “from” field shows the Ethereum address of the function caller which is the manufacturer in this case and it also shows the name and address of the function in the “to” field. Moreover, the gas, transaction, and execution costs are displayed in the following three fields respectively. Furthermore, the hash of the function call and its input are displayed in the next two fields. Finally, the “logs” field shows the emitted event which in this case declares that the container is ready for delivery and it also shows the Ethereum address of its manufacturer.
• **StartDelivery**: The distributor is the only actor in the network that is allowed to execute this function which emits an event declaring the start of the delivery process. Figure 10 shows a successful execution of the function along with its logs and events. The “from” field shows the Ethereum address of the distributor which can be also verified from Table 1. Moreover, the “to” field shows the name and address of the called function. Furthermore, the gas, transaction, and execution costs are displayed in the following three fields respectively. In addition to that, the hash of the executed function and the input are shown in the following two fields. Also, the function only logs events and has no decoded input nor decoded output. Finally, the “logs” field shows the emitted event which declares the start of the delivery process and displays the Ethereum address of the function caller who is the distributor in this case.
• **EndDelivery**: Similar to the *StartDelivery* function, this function is only executable by the distributor where an event is emitted to declare the end of the delivery process and the smart container has arrived at the vaccination center. Figure 11 shows the logs and transactions of the function. The “from” field shows the Ethereum address of the distributor and the “to” field shows the function name and its address. Furthermore, the gas, transaction, and execution costs are displayed respectively in the following three fields. Moreover, the hash and input of the function are displayed in the next two fields. Further, the “decoded input” and “decoded output” fields are empty because the function takes no input and returns nothing to its executor. Finally, the “logs” field shows the emitted event which declares the end of the delivery process and it also shows the Ethereum address of the distributor.
• **ReceiveContainer**: This function deals with the reception of the smart container by the Vaccination Center which is the only actor in the network that is eligible to execute it. An event is emitted when the function is executed successfully declaring that container has been received by the vaccination center. Figure 12 shows the logs and transactions of successful execution of the function. The “from” field shows the Ethereum address of the Vaccination Center which can be verified from Table 1 and the “to” field shows the name and address of the called function. Moreover, the gas, transaction, and execution costs are displayed respectively in the following three fields. In addition to that, the hash and the input of the function are shown respectively in the next two fields. Besides, there are no decoded input nor output because the function doesn’t take an input and returns nothing to its executor. Finally, the “logs” field displays the emitted event which declares the container reception by the Vaccination Center.
• **violationOccurrence**: This function is critical for violations monitoring where the smart container is the only entity in the Ethereum network that is capable of executing it. A signal is sent from the smart container to the smart contract whenever a violation occurs and the delivery process is aborted. Moreover, an event is emitted to notify all participants about the violation. Figure 13 shows the logs and transactions of successful execution of the function in the case of a temperature violation. The “from” field shows the Ethereum address of the smart container which can be verified from Table 1 and the “to” field shows the function name and address. The following three fields show gas, transaction, and execution costs respectively. Moreover, the hash and input of the function are displayed in the following two fields. Furthermore, this function has a decoded input which it takes from the smart container where the violation type and value are assigned. Finally, the “logs” field shows the emitted event which declares the occurrence of a violation which is temperature violation in this case along with its value. Other violations will go through a similar process and the only difference will be in the emitted event where the violation type and value will be changed accordingly.

## VI. Discussion

In this section, we present cost and security analysis for the COVID-19 vaccine delivery solution. Furthermore, we compare our approach with the existing solutions. Additionally, we discuss the proposed solution from the generalization point of view to show its scope and applicability.

### A. Cost Analysis of the Blockchain-Based Solution

The execution of transactions on the Ethereum blockchain costs *gas* which is needed to transmit the transactions to the Ethereum blockchain. The main gas costs come from the execution and transaction. The estimation of gas costs is done through the REMIX IDE platform. The execution cost refers to the cost of executing functions within the smart contract. On the other hand, the transaction cost refers to the cost of deploying the smart contract on the Ethereum blockchain and data transaction to the Ethereum blockchain.

Table 2 presents the gas costs of the smart contract functions which are presented in both gas and fiat currency (USD). The gas prices vary over time and the ones used in the analysis are accessed on Feb 13, 2021, via the ETH gas station [29]. An average gas price of 139 Gwei and an Ethereum price of 1862 USD are used in the cost analysis.

**TABLE 2:** Gas Costs of the Smart Contract Functions

Function Caller	Function Name	Transaction Gas	Execution Gas	Cost in USD
Any Participant	containerManufacturer	22328	1056	6.05
Manufacturer	distributorRegistration	44820	22140	17.33
Manufacturer	CreateContainer	45266	23994	17.93
Distributor	StartDelivery	30301	9029	10.18
Distributor	EndDelivery	30339	9067	10.20
Vaccination Center	ReceiveContainer	30201	8929	10.13
Smart Container	violationOccurrence	30850	9194	10.36

Table 2 shows that the cost of the tracking functions are the lowest because they don’t require the smart contract to store anything on-chain; whereas, the *distributorRegistration* costs a little bit more because it requires input from the manufacturer which is then stored on-chain. It also involves the manipulation of a mapping variable. An important thing to note here is the high cost of the *CreateContainer* function. Although it is very similar to the other delivery functions, this can be explained by the fact that the *CreateContainer* function will store the “State” variable for the first time that requires allocating memory space to it, and thus adding additional gas cost; whereas, other delivery functions will only need to update that variable which is an operation that does not cost as much as storing for the first time [33].

The gas fees are part of Ethereum which are the cost for mining nodes to execute transactions. The main issue with those fees is that they are not constant and they fluctuate based on the network congestion. The high Ethereum gas price is an issue in the Ethereum platform that can force developers to look for alternatives to build and design their solutions. zkSync is a trustful, secure, and user-centric protocol that is used to scale payments and smart contracts on the Ethereum blockchain. This solution promises to reduce the high costs of transactions on the Ethereum blockchain to a small fraction [34]. Another solution to this problem would be the use of a private blockchain where no fees are charged for transactions. For example, a private Ethereum blockchain can be set up where only trusted nodes can be added to the network. In addition to that, the gas price that miners accept can be modified to be zero which allows nodes to use smart contracts without worrying about transaction fees. Moreover, private blockchains prioritize immutability and efficiency over anonymity and transparency. However, transparency can still be achieved among the authorized entities only where the on-chain stored information is both visible and secure to those entities [35]

### B. Security Analysis of the Blockchain-Based COVID-19 Vaccine Delivery Solution

Different security aspects such as integrity, accountability, authorization, and availability are considered to perform the evaluation. In addition to that, resilience against Man-in-the-middle (MITM) attacks is briefly discussed.

• **Integrity:** The proposed blockchain-based COVID-19 vaccine delivery system allows its users to track, trace, and monitor the IoT-Enabled smart containers while they are being shipped which provides high integrity. This is implemented in the solution in an event-based approach where every transaction is recorded and logged on-chain. Moreover, the smart container records any violation on-chain in real-time.
• **Accountability and Authorization:** The implemented solution assigns the different participants to specific roles by using the *Modifier* feature of the Ethereum smart contract. Since transactions are recorded on an immutable ledger, all participants will be held accountable for their actions. For example, if wrong details are provided regarding the smart container, the manufacturer will be responsible for it. Another example would be the occurrence of any mistake during the delivery process where the distributor is the one who is responsible for it.
• **Availability:** The Ethereum blockchain is decentralized and distributed in nature. Therefore, all of the smart contract’s logs and transactions will be recorded by all the participating nodes. This property allows the blockchain network to remain active and keep running even if a node fails at some point. The only case that would bring the network down is when all nodes fail which is extremely unlikely in a distributed network.
• **MITM Attacks:** MITM attacks are not possible in a blockchain network because transactions need to be signed by its sender’s private key, and any attempt to change the content of the transaction will require the intruder to sign it by the sender’s private key which is not possible. This feature is essential for the success of the COVID-19 vaccine delivery system because it ensures that all the information is provided by trusted entities only.

### C. Smart Contract Security Analysis

**FIGURE 14.**
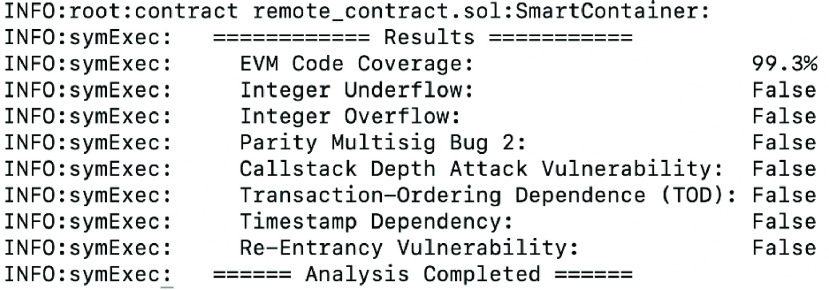
Smart contract vulnerability analysis.

Security analysis is performed on the Ethereum smart contract to test its security against some vulnerabilities; namely, integer overflow, integer underflow, parity multisig bug, callstack depth attack, transaction-ordering dependence (TOD), timestamp dependency, and re-entrancy. The Oyente tool is used to perform the desired security analysis. The tool runs on Linux operating system and it checks solidity codes for any vulnerabilities [30]. Figure 14 presents the output of the Oyente tool and it shows that there are no existing vulnerabilities within the deployed smart contract which indicates that the smart contract is robust and highly secured.

### D. Comparison With the Existing Solutions

We compare our solution with the existing non-blockchain and blockchain-based solutions proposed for vaccine delivery as can be seen in Table 3 and Table 4.

**TABLE 3:** Comparison With the Existing Non-Blockchain Based Solutions

	VVM [19]	30DTR [20]	Ouzayed et al. [15]	SenseAware ID [17]	Proposed Solution
Decentralized	No	No	No	No	Yes
Integrity	No	No	No	No	Yes
Security	No	No	No	No	Yes
Traceability	Yes	Yes	No	No	Yes
Violation Detection	Yes	Yes	Yes	Yes	Yes
Violation Detection in Real-Time	No	Yes	Yes	Yes	Yes
Accountability	No	No	No	No	Yes

**TABLE 4:** Comparison With the Existing Blockchain-Based Solutions

	Our Solution	Yong et al. [26]	Tian [23]	StaTwig [22]
Blockchain Platform	Ethereum	Ethereum	NA	NA
Mode of Operation	Public Permissioned	Public Permissioned	NA	NA
Off-Chain Data Storage	Yes	No	No	No
Tracing	Yes	Yes	Yes	Yes
Real-Time Tracking	Yes	No	No	No

The existing non-blockchain based solutions were compared based on important parameters; namely, decentralization, integrity, security, traceability, violation detection, violation detection in real-time, and accountability. Decentralization, which refers to how many entities are in control of the stored data, and the comparison shows that non-blockchain based solutions all have their data stored centrally that allows a single entity to control and manipulate the data; whereas, our proposed solution is decentralized and no single entity has control over the data. Integrity, which refers to the correctness, consistency, and accuracy of the stored data, is another important feature that is missing in non-blockchain solutions because a single entity is controlling the database and there is no guarantee that no manipulation will occur; whereas, our proposed solution inherently has this feature because it uses blockchain technology. Security, which refers to how secure the system is against intruders, is a feature that is not guaranteed in non-blockchain solutions because having a centralized system makes it easier for intruders to attack the system; whereas, in our proposed solution, data is stored in a decentralized way that makes it almost impossible for unauthorized entities to make changes. Tracing is an important feature that allows the users to trace the origin of the vaccines and also to inspect all actions throughout the delivery process. Unlike the existing solutions, only VVM, 30DTR, and our proposed solution offer traceability features. Violation detection allows the users to get notified about violations. All solutions are capable of detecting violations in real-time except the VVM that does not send any notifications to the users. Accountability is an important feature that accounts for one’s actions. In our proposed solution, all users must be registered and authorized and all of their actions are stored on an immutable ledger that prevents them from denying them. On the other hand, non-blockchain solutions do not provide accountability because there is no proper registration.

Table 4 compares different blockchain-based solutions that address the trace and track issue in supply chains. Our proposed solution uses the Ethereum blockchain that operates with a public permissioned mode. Moreover, it has off-chain data storage, and it is capable of performing both tracing and real-time tracking. Yong et al [26] proposed an Ethereum blockchain-based solution that uses public permissioned mode. However, the solution does not offer off-chain storage and real-time tracking feature. Tian [23] proposed a blockchain-based tracing solution for the agriculture food supply chain; however, the solution has no technical work and it only suggests how blockchain can be used for that purpose. Finally, StaTwig [22] is another blockchain-based solution for the vaccine supply chain that promises to provide tracing for the delivered vaccines; however, the solution is still under development and no technical work is currently available. Based on this comparison, the used methodology in our solution shows some differences compared to the existing solutions. For example, the previous solutions were attempting to trace the supply chain; whereas, our solution provides both tracing and real-time tracking to notify all concerned entities about any violation as it occurs, thereby improving the overall performance of the system. Furthermore, our solution is integrated with off-chain storage that allows authorized users to upload large files off-chain, thereby reducing costs.

### E. Generalization

The proposed blockchain-based solution demonstrates how blockchain technology can be leveraged to track, trace, and monitor COVID-19 vaccines while they are being delivered and distributed. The smart contract code is customized to fit and fulfill the needs of the COVID-19 vaccine delivery system. However, it can be customized to other supply chains.

Other supply chains will contain very similar components to the ones proposed in this paper. However, the main difference will be in the TRU which might require special conditions while it is being shipped. For example, the food supply chain will require monitoring of the food products but they need to be handled differently [27]. In our case, the COVID-19 vaccine requires ultra-low temperature; whereas, food products require temperature levels that very rarely reach those of the COVID-19 vaccine. Therefore, the major modifications will be on the smart container where it has to be prepared to maintain the needed temperature level and equipped with monitoring sensors that can detect violations. Based on that, the smart contract can be slightly modified to accommodate the needs of the new supply chain. Specifically, the *violationOccurrence* function will need to be triggered based on the new violation values of the targeted supply chain.

Figure 2 can further clarify the needed modifications for the new supply chain. First, the actors will most likely be different and there might additional registration needed for them. Therefore, the registration function in the smart contract will need to be extended to accommodate the additional actors. Moreover, off-chain storage might not be necessary if the supply chain doesn’t contain large-sized data that needs to be stored or accessed. Finally, algorithms can be easily modified to fit the functions needs of the new supply chain because other supply chains follow similar algorithms [28].

## VII. Conclusion

In this paper, we have addressed important issues related to tracking, tracing, and monitoring of COVID-19 vaccines’ distribution and delivery. We proposed an Ethereum blockchain-based approach to enable the distribution and delivery process of COVID-19 vaccines in a manner that is decentralized, traceable, transparent, reliable, auditable, secure, and trustworthy. We developed smart contracts to automate the process of recording and logging events related to the COVID-19 vaccines’ distribution and delivery, thereby ensuring data provenance. We connected the Ethereum blockchain to off-chain storage to alleviate the limited data storage issue. We presented architecture details, system components, and algorithms along with their implementation, testing, and validation details. We analyzed costs associated with the execution of functions within the smart contracts to show our solution’s affordability. We conducted the security analysis for the smart contract to check its robustness against well-known vulnerabilities. Additionally, we presented the general security analysis to show that the proposed approach is secure enough against malicious attacks. We compared our solution with the existing non-blockchain and blockchain-based solutions. Our proposed solution is generic and can be adapted into any type of vaccines’ traceability and monitoring scenarios with minimal modifications and efforts. Although the proposed solution has addressed some important issues, it still has some limitations. For example, the use of a public permissioned Ethereum blockchain will require the users to spend Ether to execute their functions, and given how volatile the gas price is, it can be difficult to estimate the cost of executing functions. Moreover, having immutability in our proposed solution can be a double-edged sword because any human error will be permanently stored and it cannot be altered. In addition to that, the public Ethereum blockchain uses Proof-of-Work (PoW) consensus algorithm that poses scalability challenges. Finally, if different blockchain solutions are adopted and are built on different blockchain platforms, it can lead to interoperability issues. In the future, the proposed solution can be improved by doing the following:

• Building a private permissioned blockchain that can be configured to eliminate gas prices.
• In a private Ethereum blockchain, different consensus algorithms such as Proof of Stake (PoS) and Proof of Authority (PoA) can be used to improve scalability.
• To further improve privacy, the Quorum platform can be used to ensure that transactions between entities can be only viewed by those entities and nobody else.
